# Semiautomatic, Quantitative Measurement of Aortic Valve Area Using CTA: Validation and Comparison with Transthoracic Echocardiography

**DOI:** 10.1155/2015/648283

**Published:** 2015-06-29

**Authors:** V. Tuncay, N. Prakken, P. M. A. van Ooijen, R. P. J. Budde, T. Leiner, M. Oudkerk

**Affiliations:** ^1^Center for Medical Imaging North East Netherlands (CMI-NEN), Department of Radiology, University of Groningen, University Medical Center Groningen, P.O. Box 30001, 9700 RB Groningen, Netherlands; ^2^Department of Radiology, University of Groningen, University Medical Center, P.O. Box 30001, 9700 RB Groningen, Netherlands; ^3^Department of Radiology, University Medical Center Utrecht, P.O. Box 85500, 3508 GA Utrecht, Netherlands

## Abstract

*Objective*. The aim of this work was to develop a fast and robust (semi)automatic segmentation technique of the aortic valve area (AVA) MDCT datasets. *Methods*. The algorithm starts with detection and cropping of Sinus of Valsalva on MPR image. The cropped image is then binarized and seed points are manually selected to create an initial contour. The contour moves automatically towards the edge of aortic AVA to obtain a segmentation of the AVA. AVA was segmented semiautomatically and manually by two observers in multiphase cardiac CT scans of 25 patients. Validation of the algorithm was obtained by comparing to Transthoracic Echocardiography (TTE). Intra- and interobserver variability were calculated by relative differences. Differences between TTE and MDCT manual and semiautomatic measurements were assessed by Bland-Altman analysis. Time required for manual and semiautomatic segmentations was recorded. *Results*. Mean differences from TTE were −0.19 (95% CI: −0.74 to 0.34) cm^2^ for manual and −0.10 (95% CI: −0.45 to 0.25) cm^2^ for semiautomatic measurements. Intra- and interobserver variability were 8.4 ± 7.1% and 27.6 ± 16.0% for manual, and 5.8 ± 4.5% and 16.8 ± 12.7% for semiautomatic measurements, respectively. *Conclusion*. Newly developed semiautomatic segmentation provides an accurate, more reproducible, and faster AVA segmentation result.

## 1. Introduction

Aortic stenosis (AS) is the most common valvular heart disease in the developed countries, affecting 3 percent of the population older than 65 years. It causes higher morbidity and mortality than any other cardiac valve disease [1]. AS is defined as narrowing of the aortic valve opening, which reduces blood flow from the heart into the aorta. The normal size of the aortic valve area (AVA) at maximum opening of the valve is 3 to 4 cm^2^ [2]. When the AVA decreases below 1 cm^2^, AS is considered to be severe [3]. For severe AS, valve replacement is the only effective treatment. However, a sizeable fraction of patients are at high risk for postoperative mortality and may refuse surgery or cannot undergo surgery due to comorbidity [4]. Recently, transcatheter aortic valve replacement (TAVR) techniques have been developed to provide less invasive treatment for those patients [5–11]. In management of AS, the timing for surgical treatment is very important. Late treatment may lead to an increase in the transaortic pressure gradient, myocardial pressure overload, and eventually left ventricular (LV) hypertrophy and increased LV wall thickness [12]. Visualization of the AVA is used to determine the threshold for invasive treatment and to obtain preoperative information about the aortic dimensions and proximity to other important structures such as the coronary arteries.

Different imaging modalities have been used and compared for measuring the AVA [13–23]. Initially, catheterization was the standard method for evaluating AVA, but in time its usage decreased due to its being an invasive modality and technical limitations. Alternatively, the 2D echocardiographic continuity equation, which currently is the most common tool to derive the AVA, was used to measure the AVA. However, this technique underestimates AVA since it assumes that the left ventricular outflow tract (LVOT) has circular geometry and that flow through the LVOT is laminar and uniform [24, 25]. Bruder et al. showed a strong correlation between the AVA determined by echocardiography and MRI [20] indicating that MRI can also be used to determine AVA. However, MRI is contraindicated for patients with metal implants or claustrophobia. Moreover MRI has lower spatial resolution in comparison to CT [12]. More recently, development of ECG-gated multidetector computed tomography (MDCT) has led to further improvements in cardiac imaging [21], and CT is now also regarded as a reliable method to measure the AVA [26]. The latest developments in dual source and 320-slice CT enable high temporal resolution acquisition and obtain sufficient image quality at high spatial resolution in almost every patient throughout the cardiac cycle. However, streaking and blooming artifacts due to heavy calcification of the aortic valve leaflets or the aortic root which hamper visualization and analysis of the AVA can still occur.

Delgado and Bax suggested that 3D planimetric measurement of the AVA by MDCT images may provide more reliable information on the assessment of AVA in comparison with echocardiography [27]. However, planimetric measurement of AVA is currently performed manually by the radiologist using standard 3D visualization and measurement software, which is time consuming and introduces user dependence and intra- and interobserver variability [28].

The aim of this study was to develop and validate a (semi)automatic segmentation technique of the AVA and to compare manual and semiautomatic measurements with the Transthoracic Echocardiography (TTE) results. Our goal is to reduce the user dependency and time spent on measurements and to enable reproducible and accurate measurement of AVA on MDCT datasets.

## 2. Materials and Methods

### 2.1. Experimental Design

In this study multiphase CT scans of 25 patients (15 female, mean age 82.84 ± 5.16 years) were used. All of the patients had moderate to severe aortic stenosis and underwent TAVI at a tertiary referral center. All subjects underwent CT scanning and TTE.

Informed consent requirement was waived by the local IRB because of the retrospective nature of this study without additional burden to the patients involved.

The maximum aortic valve opening phase was selected visually for all patients. A stack of reformations was obtained after centering the axis of the multiplanar reconstruction (MPR) at the level of aortic valve and then changing the orientation of the plane perpendicular to the LVOT. The preselected slices were segmented both manually and semiautomatically by two independent observers 1, a biomedical engineer with more than 5 years of experience, and 2, a cardiac radiologist with almost 10 years of medical imaging experience. Observer 1 repeated the measurement 1 day after in order to determine intraobserver variability. Manual and semiautomatic measurements were compared with the current reference standard Transthoracic Echocardiography (TTE) with regard to AVA. Time spent for measurements was recorded for manual and semiautomatic segmentations.

### 2.2. Transthoracic Echocardiography

TTE was performed as part of the routine workup of the patient. TTE derived AVA measurements were obtained from the clinical patient records. The velocity in the left ventricular outflow tract and at the level of the aortic valve and the LVOT diameter were measured. From these measurements the AVA was calculated using the continuity equation.

### 2.3. Multislice Computed Tomography

Image acquisition of the retrospectively ECG-gated CTA of the thoracoabdominal aorta was performed on a multidetector 256-slice CT (Brilliance iCT, Philips Healthcare, Best, The Netherlands). An ECG trace was recorded during the procedure. The region of acquisition ranged from above the aortic arch to the groin. Based on a locator image, a circular region of interest was drawn within the descending aorta. Nonionic iodinated contrast material (Ultravist, 300 mg iopromide per mL, Schering Nederland BV, Weesp, The Netherlands) was injected intravenously. As soon as the descending aorta reached a density of 125 Hounsfield units (HU) within the region of interest, the patient was instructed to maintain a breath hold. Seven seconds later, image acquisition started in a craniocaudal direction with concurrent ECG trace recording. The following parameters were used: detector collimation 128 × 0.625 mm; pitch 0.30; matrix size 512 × 512. Tube voltage and tube current-time product depended on the patient's weight and were 100 kVp and 300 mAs, respectively, for patients <70 kg, and 120 kVp and 250 mAs, respectively, for patients ≥70 kg.

### 2.4. AVA Segmentation Algorithm

**Figure 1:**
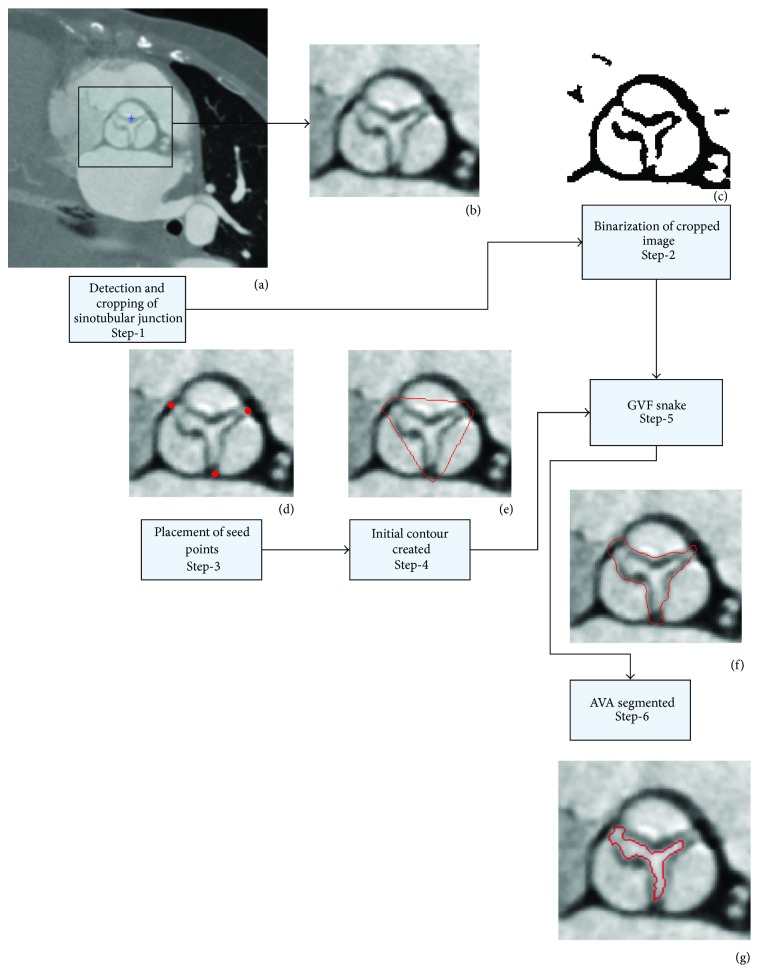
The flowchart of the general algorithm.

The segmentation algorithm (Figure 1) starts with the detection of the Sinus of Valsalva (SOV) on the MPR image. Once the SOV is detected, the region covering the SOV is cropped from the whole image (Figure 1(a)). The cropped image (Figure 1(b)) is binarized by adaptive thresholding (Figure 1(c)). The user places seed points (Figure 1(d)) to create an initial contour (Figure 1(e)) covering the aortic valve opening. The contour moves towards the edge of the aortic valve opening automatically (Figure 1(f)). The contour covers the pixels from the edge of the opening area and also the AVA opening area. The pixels of the opening area are selected and the number of pixels is multiplied by the pixel size to determine the AV opening area (Figure 1(g)).

#### 2.4.1. Detection and Cropping of the Sinus of Valsalva

**Figure 2:**
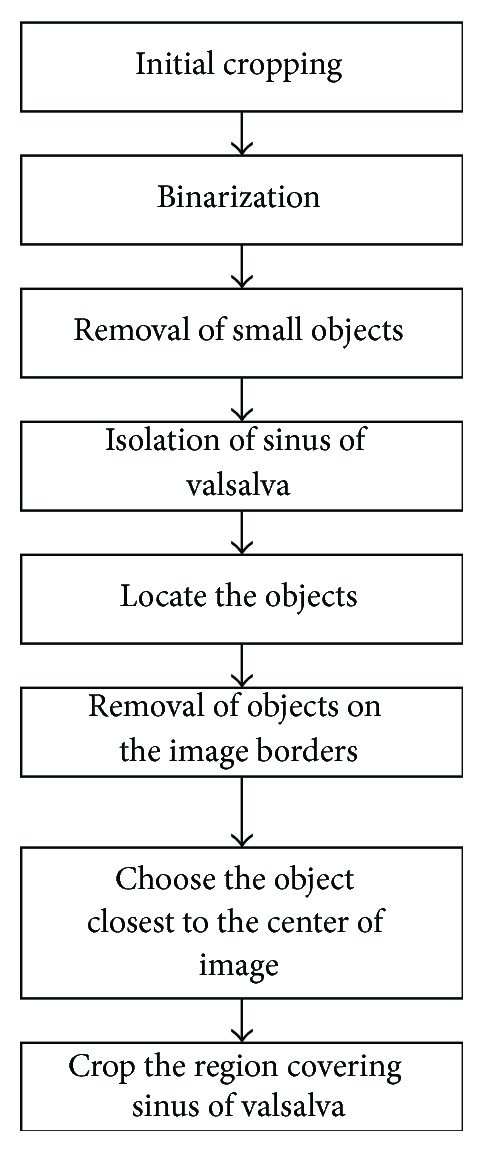
The flowchart of the SOV detection and cropping algorithm.

Image cropping was used to reduce computation time. In the object detection the object size, shape, location, and orientation play major roles. Since the SOV is located in the central part of the MPR image, detection and cropping of this region begin with a preliminary cropping operation, which covers the most of the central part of the MPR image. After the initial cropping, the grayscale image is binarized using global thresholding with a threshold level based on the histogram of the image. The binary image contains only white (object) and black (background) pixels, which enables detection of the objects in the image and facilitates the use of morphological operations. Following binarization, objects smaller than 700 pixels were removed. Secondly the SOV was disconnected and isolated. The SOV is located in the central part of image, such that objects on the border of the image were removed. After detection of the SOV, the region covering it was cropped from the image (Figure 2). All following operations were performed on the cropped image.

#### 2.4.2. Segmentation of AVA

**Figure 3:**
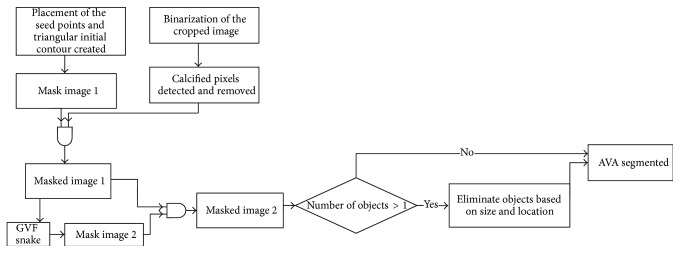
The flowchart of the AVA segmentation algorithm.

The flowchart in Figure 3 shows the details of the segmentation of the AVA. The main segmentation tool is the gradient vector flow (GVF) snake [29]. The snake algorithm is an active contour, which moves to the edges of the object in order to reach the boundaries of the object. In the binarized images (described above) the edges are clearer and the active contour can move towards the object boundaries easier compared to grayscale images. The user places three seed points on the grayscale image where the cusps are connected to each other to create the initial contour for the GVF snake. This initial contour creates a mask image, which is used on the binarized image. The active contour shrinks to cover the AVA region. However, the GVF snake result overestimates the AVA. Therefore this GVF snake contour is used to mask the image again. In case the resulting double-masked image contains more than 1 object, the size and location of these objects were determined. Objects smaller than 40 mm^2^ and the object most distant from the center were removed. The remaining object was identified as the AVA.

#### 2.4.3. Detection and Removal of Calcification

**Figure 4:**
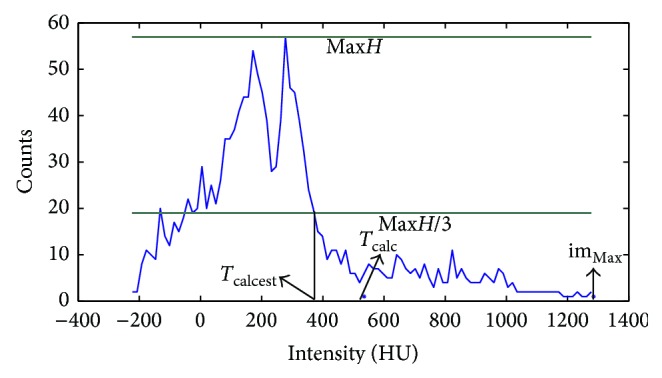
The histogram of the grayscale image.

Aortic valve calcification is very common in a population with AS. In order to segment the AVA properly one must first detect the calcifications and then exclude the calcified areas from the AVA region. Since a contrast agent was used in the CT scans we cannot use the fixed 130 HU threshold to detect pixels in calcified areas. We therefore developed an algorithm to determine the threshold of calcified pixels, consisting of the following 5 steps:

(1) Calculation of the histogram (Figure 4) and determination of the index number (index) of the maximum pixel intensity (im_Max_).
(2) Calculation of the maximum histogram value (Max*H*).
(3) Decreasing the index until reaching Max*H*/3 and setting the corresponding intensity level as the initial estimation (*T*_calcest_) for calculation of calcium threshold (*T*_calc_).
(4) Determination of the dynamic range of the image.
  (i) Starting from the first bin of the histogram, the amount of pixels in each bin was counted until reaching half of the total number of pixels.
  (ii) The index number of the histogram bin where the algorithm stopped corresponds to the dynamic range (DR) of the image.
(5) Calculation of the calcium threshold (*T*_calc_) for DR > 0.7*∗*im_Max_ (brighter images) by (1) and *T*_calc_ for DR < 0.7*∗*im_Max_ was calculated by (2):
  (1) $$\begin{array}{c} T_{\text{calc}}=T_{\text{calcest}}+\left({\text{im}}_{\mathrm{Max}}-T_{\text{calcest}}\right)*0.5, \end{array}$$
  (2) $$\begin{array}{c} T_{\text{calc}}=T_{\text{calcest}}+\left({\text{im}}_{\mathrm{Max}}-T_{\text{calcest}}\right)*0.2. \end{array}$$

**Figure 5:**
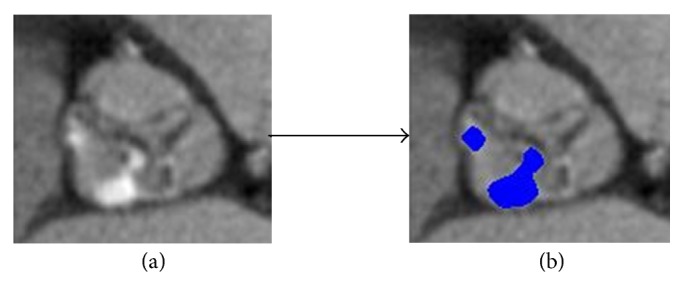
Example of the calcium detection algorithm.

An example is given in Figure 5. The calcified pixels on grayscale image (Figure 5(a)) are detected and given a blue color (Figure 5(b)).

### 2.5. Computation Time

Computation time was defined as the time between the visualization of the final cropped image and the display of the measurement of the AVA size. For manual measurements it included the time required for the user to trace the orifice perimeter and the calculation of the selected area. For the semiautomatic measurements it included the selection of the seed points by the user and the computation of AVA based on the semiautomatic segmentation results. The time was measured internally by the developed software tool and displayed when the AVA size measurement was finished.

### 2.6. Validation and Statistical Analysis

Relative differences between the measurements were calculated to determine (1) the intraobserver variability of the semiautomatic measurements and (2) the intraobserver variability of the manual measurements. Relative difference was calculated as follows:

(3) $$\begin{array}{c} \text{Relative difference}=\frac{\text{Absolute difference}*100}{\text{Mean of the measurements}}. \end{array}$$

Differences between TTE and MDCT manual and semiautomatic measurements were assessed by Bland-Altman plots. Statistical analyses were performed using IBM SPSS Statistics version 20.0.0.1 (SPSS Inc., Chicago, USA).

## 3. Results

### 3.1. Segmentation Results

**Figure 6:**
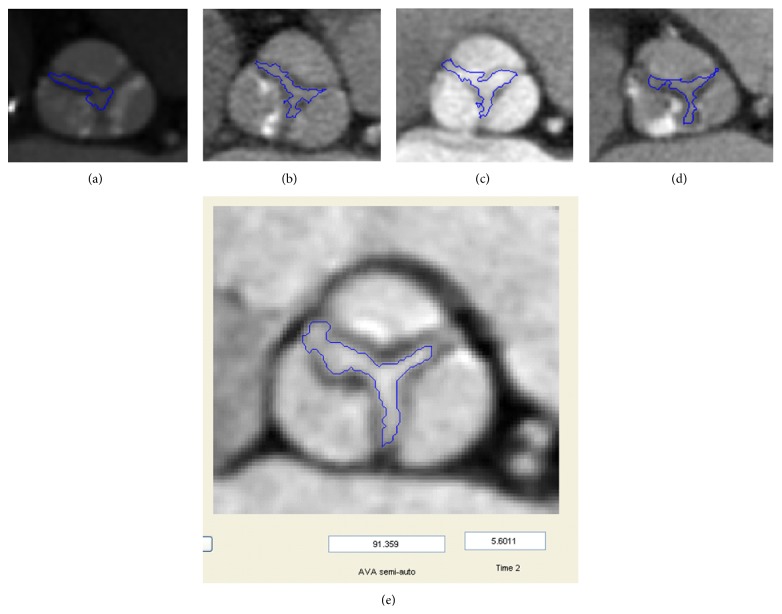
Results of semiautomatic measurements on various images (a–d). Result on graphic user interface (e).

Aortic valve areas as measured manually and semiautomatically are listed in Table 1. Sample results of the semiautomatic segmentation are given in Figure 6. Semiautomatic segmentation of AVA was achieved successfully for both calcified (Figures 6(a), 6(b), and 6(d)) and noncalcified aortic valves (Figure 6(c)). The output result of a segmentation and computation time for a sample image is given in Figure 6(e) showing one part of the graphic user interface.

### 3.2. Computation Time

The computation times of both observers were shorter for the semiautomatic measurements. Manual measurements took 18.85 ± 5.66 seconds and 16.69 ± 3.69 seconds for observer 1 and observer 2, respectively. Semiautomatic measurements were 5.06 ± 0.72 (observer 1) and 6.68 ± 1.79 seconds (observer 2).

### 3.3. Observer Variability

Differences in intraobserver variability of manual and semiautomatic measurements are listed in Table 2. Both intra- and interobserver variability were lower for semiautomatic measurements.

### 3.4. Comparing Manual and Semiautomatic Measurements with TTE

**Figure 7:**
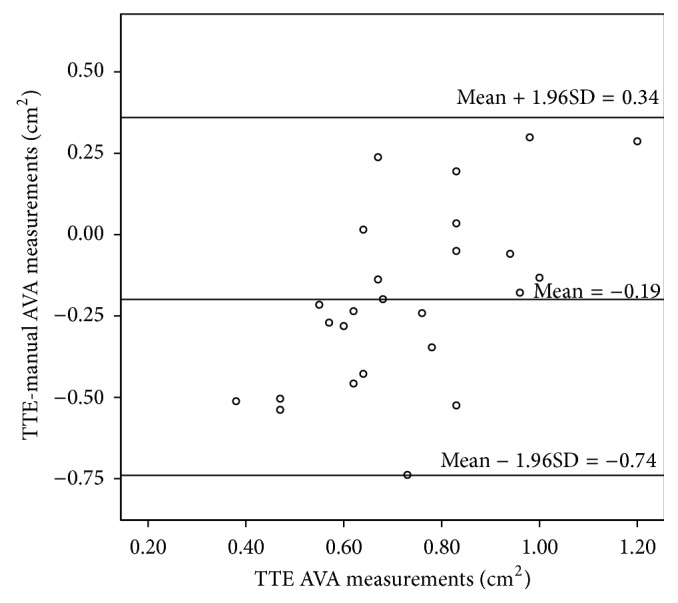
Bland-Altman plot between the TTE and manual AVA measurements.

**Figure 8:**
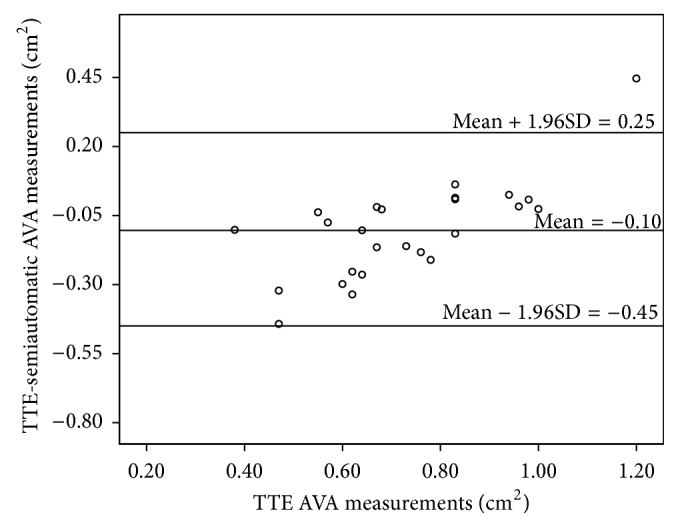
Bland-Altman plot between the TTE and semiautomatic AVA measurements.

Comparisons of the manual and semiautomatic measurements with TTE results were performed using Bland-Altman plots; mean difference between TTE and MDCT results was −0.19 (95% CI: −0.74 to 0.34) cm^2^ for manual and −0.10 (95% CI: −0.45 to 0.25) cm^2^ for semiautomatic measurements (Figures 7 and 8). The differences were significantly different from 0 (*p* = 0.001 for manual and *p* = 0.007 for semiautomatic measurements) indicating a bias. Both mean difference and the confidence interval are smaller in the comparison of TTE and semiautomatic measurements which indicates that semiautomatic measurements are closer to the TTE measurements than to the manual measurements.

## 4. Discussion

### 4.1. Research Summary

In this study we propose a semiautomatic segmentation technique to measure the AVA and compared it with the manual segmentation using TTE measurements as the reference standard. The focus of the study was to investigate whether the repeatability and reproducibility of the AVA measurements can be improved with the semiautomatic segmentation along with an improvement in computation time. First of all, our results show that semiautomatic measurements are closer to the reference TTE measurements. Furthermore the intra- and interobserver variations are lower for the semiautomatic measurements compared to manual measurements. Finally semiautomatic measurements are more than 10 seconds faster than the manual measurements.

### 4.2. Previous Studies and Current Study

TTE is currently the most widely used imaging modality to measure the AVA. The continuity equation, which is used to calculate the AVA based on 2D TTE data, assumes that the LVOT has a circular shape. A recent study showed that this assumption might cause underestimation of the AVA [30]. TTE was compared to CT in several studies. Larsen et al. observed 6% and 16% intra- and interobserver variability for MDCT measurements on patient with broad severity of AS. Meanwhile the intra- and interobserver variability were 13% and 19% for the TTE measurements [31]. In our study the interobserver variability of semiautomatic measurements was 16% in the measurements on the patients with severe AS. Lembcke et al. conducted a study with 160 patients using 64-MDCT and TTE. They found 0.17 ± 0.24 cm^2^ mean difference between MDCT and TTE measurements [32]. In our study we observed 0.19 ± 0.27 cm^2^ and 0.10 ± 0.18 cm^2^ mean differences in the comparisons of TTE with manual and semiautomatic MDCT measurements, respectively.

Even though (semi)automatic quantification of the aortic root dimensions such as aortic annulus, Sinus of Valsalva, and sinotubular junction using CT data has already been available in the literature [33] there is a paucity of data about (semi)automatic quantification of the AVA using CT images. Previous research already showed that echocardiography underestimates that the AVA and CT planimetric measurements are closer to the real AVA. Moreover CT is the modality used for measuring the aortic valve calcium score which associated with AS. All of these reasons make CT the method of choice. However, planimetric CT measurement of the AVA is currently performed manually, which is user dependent and time consuming. Our results demonstrate the feasibility of developing an algorithm for semiautomatic quantitative measurements of AVA in order to reduce observer variability and the time spent on the measurements. Moreover this technique is shown to also work on the target population of AS patients with a significant calcium load. The calcified regions should be detected and the opening area should be segmented excluding the calcified area. A calcification threshold is needed in order to detect the pixels belonging to the calcified region. However, in virtually all CT scans made for preoperative evaluation in patients with aortic stenosis a contrast agent is injected which makes it impossible to set a fixed calcification threshold. To overcome this issue, an algorithm was developed to calculate the calcification threshold for each CTA image individually.

The ultimate goal of fully automatic user independent segmentation was not achieved and user selection of three seed points is still required in the semiautomatic segmentation. Main reason for this is that the image quality with the current CT technology does not allow making the AVA segmentation fully automatic due to unclear object (AV) boundaries in some cases. The GVF snake was the method of choice since the snake algorithm works in cases where some parts of the object boundaries are not clear. A possible solution to make the segmentation less user dependent could be to develop an algorithm which can detect parts of the AVA boundaries (semi)automatically and interpolate the rest of the object boundary. Further developments in CT technology with higher spatial resolution and less calcium artifacts might also help to achieve the goal of fully automatic segmentation of the AVA. Our results show that CT based AVA segmentation can be achieved with less user dependence and as a result a higher reproducibility and less time consuming measurements of AVA segmentation were obtained.

### 4.3. Limitations

A possible limitation of our study was that the users were not asked to rechoose the phase and opening plane on which to measure the AVA. However, this choice was made to eliminate the user interference in the measurement results in order to really test the accuracy of the developed algorithm. Another limitation of this study is the selected patient group. We studied a relatively small sample of patients with varying delay between TTE and CT imaging and all subjects had severe AS (mean AVA smaller than 1.0 cm^2^). Future work will have to be carried out in larger cohorts containing subject with varying degrees of AS. Also, we cannot rule out that differences in AVA can be attributed to differences in area over time as opposed to difference inherent to the imaging techniques used. The time difference between the CT and ultrasound measurements was more than 100 days for 6 patients. A final limitation is having 2 different measurement techniques using the TTE and MDCT data. In MDCT measurements we had the direct measurements using the planimetric image of the AVA; on the other hand AVA was measured indirectly by the flow information gathered by the TTE. This difference between the measurement techniques led to the variation between the TTE and MDCT manual and semiautomatic measurements. Therefore further work required to determine what the clinical followup should be based on MDCT measurements.

### 4.4. Implications

Studies comparing the use of CT and echocardiography found that CT can be an alternative to the current gold standard echocardiography in the quantification of AVA [26, 27]. Our study has some implications in semiautomatic quantification of AVA on the CT images. First of all the intra- and interobserver variability of semiautomatic measurements are better than the manual measurements. These results imply that the variation caused by the user interaction is decreased by using the semiautomatic software, which is desirable for quantitative assessment of medical images. Moreover semiautomatic software provides a faster calculation of the AVA in comparison with the manual measurements. Faster measurements decrease the workload. The comparison of manual and semiautomatic CT measurements with the current standard TTE measurements revealed that semiautomatic measurements are closer to the TTE measurements. If the standard modality for measuring the AVA will switch from echocardiography to CT, semiautomatic measurements can serve as a better option in comparison to the manual measurements due to the smaller difference between the TTE and semiautomatic measurements.

## 5. Conclusion

In this study a semiautomatic segmentation technique that can be used in AVA segmentation is proposed. Based on preliminary results the algorithm provides adequate segmentation of representative images, also those including severe calcification, and provides a faster, more accurate, and more reproducible AVA segmentation compared to the currently used manual segmentation.

## Figures and Tables

**Table 1:** Manual and semiautomatic AVA measurements.

	Mean ± SD
Observer 1, manual measurements (cm^2^)	0.88 ± 0.23
Observer 1, semiautomatic measurements (cm^2^)	0.85 ± 0.15
Observer 2, manual measurements (cm^2^)	0.98 ± 0.29
Observer 2, semiautomatic measurements (cm^2^)	0.82 ± 0.18

**Table 2:** Observer variability.

	Relative difference (%)
Intraobserver variability, manual	8.4 ± 7.1
Intraobserver variability, semiautomatic	5.8 ± 4.5
Interobserver variability, manual	27.6 ± 16.0
Interobserver variability, semiautomatic	16.8 ± 12.7
